# Clinical Features and Chest Imaging as Predictors of Intensity of Care in Patients with COVID-19

**DOI:** 10.3390/jcm9092990

**Published:** 2020-09-16

**Authors:** Elisabetta Cocconcelli, Davide Biondini, Chiara Giraudo, Sara Lococo, Nicol Bernardinello, Giulia Fichera, Giulio Barbiero, Gioele Castelli, Silvia Cavinato, Anna Ferrari, Marina Saetta, Annamaria Cattelan, Paolo Spagnolo, Elisabetta Balestro

**Affiliations:** 1 Department of Cardiac, Thoracic, Vascular Sciences and Public Health, University of Padova and Padova City Hospital, 35128 Padova, Italy; ecocconcelli@icloud.com (E.C.); dav.biondini@gmail.com (D.B.); sara.lococo@aopd.veneto.it (S.L.); nicol.bernardinello@unipd.it (N.B.); marina.saetta@unipd.it (M.S.); paolo.spagnolo@unipd.it (P.S.); 2 Department of Medicine, Institute of Radiology, University of Padova, 35128 Padova, Italy; chiara.giraudo@unipd.it (C.G.); gfichera90@gmail.com (G.F.); giulio.barbiero@aopd.veneto.it (G.B.); 3 School of Medicine, University of Padova, 35128 Padova, Italy; gioelecastelli@gmail.com; 4 Division of Infectious and Tropical Diseases, Azienda Ospedaliera and University of Padova, 35128 Padova, Italy; silvia.cavinato@aopd.veneto.it (S.C.); anna.ferrari@aopd.veneto.it (A.F.); annamaria.cattelan@aopd.veneto.it (A.C.)

**Keywords:** COVID-19, outcome of severity, chest X-ray, ultrasound

## Abstract

Coronavirus disease 2019 (COVID-19) has rapidly become a global pandemic with lung disease representing the main cause of morbidity and mortality. Conventional chest-X ray (CXR) and ultrasound (US) are valuable instruments to assess the extent of lung involvement. We investigated the relationship between CXR scores on admission and the level of medical care required in patients with COVID-19. Further, we assessed the CXR-US correlation to explore the role of ultrasound in monitoring the course of COVID-19 pneumonia. Clinical features and CXR scores were obtained at admission and correlated with the level of intensity of care required [high- (HIMC) versus low-intensity medical care (LIMC)]. In a subgroup of patients, US findings were correlated with clinical and radiographic parameters. On hospital admission, CXR global score was higher in HIMCs compared to LIMC. Smoking history, pO_2_ on admission, cardiovascular and oncologic diseases were independent predictors of HIMC. The US score was positively correlated with FiO_2_ while the correlation with CXR global score only trended towards significance. Our study identifies clinical and radiographic features that strongly correlate with higher levels of medical care. The role of lung ultrasound in this setting remains undetermined and needs to be explored in larger prospective studies.

## 1. Introduction

Since December 2019, when the first cases of coronavirus disease 2019 (COVID-19) were reported, the diffusion of the severe acute respiratory syndrome coronavirus type 2 (SARS-CoV-2) has rapidly spread from the Hubei Province in China to involve up to 213 states and territories to date, reaching pandemic proportions [1]. Despite epidemiological reports showing that approximately half of the infected people are asymptomatic [1], the spectrum of respiratory manifestations may range from mild symptoms, such as dry cough, fever, and fatigue, to acute respiratory distress syndrome (ARDS), requiring admission to intensive care unit (ICU) and mechanical ventilation (MV). In this scenario, thoracic radiology plays a key role in early detection of lung involvement from COVID-19. Chest computed tomography (CT) is the technique with the highest sensitivity, but the risk of contamination and the need for a dedicated hospital organization makes CT hardly available in an emergency setting. Portable chest X-ray (CXR) and ultrasonography (US) are quicker, safer and less expensive alternatives [2]. CXR is recommended as first level assessment by several scientific societies (American College of Radiology, Society of Thoracic Radiology) in the context of the SARS-CoV-2 pandemic [3]. Predominant CXR features in patients with COVID-19 include lower lobe, peripheral, bilateral ground glass opacities (GGO) or consolidations [2], similar to other forms of viral pneumonias, such as the H1N1 strain [4]. Yet, CXR could be normal in as many as 31% cases, peaking its sensibility in patients with advanced disease [5,6,7]. In the last three decades, lung US (LUS) has become increasingly important in clinical practice, particularly in the assessment of patients with pneumonia, with sensitivity and specificity of 92% and 93%, respectively, especially when performed by experienced operators [8]. In the COVID-19 pandemic, LUS has been used in multiple centers as first radiological approach in patients with suspected pneumonia. The main ultrasound findings include multiple B-lines (separated or coalescent), peripheral consolidations and thickened pleural lines [9], which however are nonspecific and found in a number of infectious and non-infectious diseases [10]. The use of LUS and CXR in combination has the potential to facilitate the identifications of ARDS [11]. With this background, we investigated the relationship between CXR severity score on admission and the level of medical care required in patients with COVID-19. Further, we assessed the radiographic–ultrasound correlation with the aim to explore the value of ultrasound in monitoring the course of COVID-19 pneumonia.

## 2. Materials and Methods

### 2.1. Study Population and Study Design

In this longitudinal retrospective study, we identified a cohort of clinically well-characterized patients with SARS-CoV-2 infection referred to the University Hospital of Padova (Division of Infectious and Tropical Diseases, Respiratory Disease Unit and Intensive Care Unit) between March and May 2020. One hundred and two patients were included in the study (Table 1) since the diagnosis of SARS-CoV-2 infection was made based on nasopharyngeal swab positivity. Clinical and demographics data, and CXRs were obtained on admission. A subset of 25 patients (25/102, 24.5%), who were hospitalized in a low-intensity care setting, underwent a bedside LUS and a CXR in the late phase of COVID-19 pneumonia. The aim of performing LUS and CXR in parallel was to explore the relation between these two procedures.

### 2.2. Level of Care Definition

The need for invasive/non-invasive ventilation or high-flow nasal cannula (HFNC), which required admission to ICU or to the Respiratory ICU, was considered as high-intensity medical care (HIMC), while the need for low flow oxygen supplementation through nasal cannula or face mask, which required the setting of a general ward, was considered as low-intensity medical care (LIMC). The level of care could change over time based on patient’s clinical conditions. For all patients, clinical data (demographics and comorbidities), gas exchange values (FiO_2_, pO_2_ and pO_2_/FiO_2_) were collected on admission (Table 1). We have categorized the five most frequent type of comorbidities: cardiovascular diseases (CVD), respiratory diseases, metabolic diseases, autoimmune diseases and oncologic diseases. Among the metabolic comorbidities, we have considered diabetes mellitus, obesity and dyslipidemia (13%). Oncologic history mentioned the different organs affected (i.e., lung, prostate, pancreas. breast, colon).

### 2.3. Ethics Statement

This was a retrospective study on anonymized patient’s data collected from electronic medical records. The study protocol complies to the ethical guidelines of the 1975 Declaration of Helsinki and, in agreement with national regulation on retrospective observational studies, it was notified and approved by the local ethics committee (n°46430/03.08.2020) and the need for patient’s informed consent was waived.

### 2.4. Data Collection

We retrieved data on patients hospitalized for COVID-19 between March and May 2020 at the University Hospital of Padova, one of the most affected areas in North-East of Italy. We screened records of all patients admitted to our Hospital with a diagnosis of SARS-CoV2 infection.

### 2.5. Radiological Evaluation

For each patient, a single image plane CXR was available on hospital admission. Two radiologists (C.G., G.B.) with more than ten years of experience in the thoracic field, who were blind to clinical data, scored the images independently using a semi-quantitative scale. This represented a modification of previously reported scoring systems that allowed to evaluate the extension of ground glass opacities (GGO) and consolidation (CO) [6,12,13]. For each lung lobe, the two radiologists assessed the extent of GGO and CO using the following scale: 0 (normal), 1 (up to 30% of the lobe involved), 2 (30% to 60% of the lobe involved), and 3 (more than 60% of the lobe involved). The sum of the scores for each lung lobe and a final value of GGO and CO score for each patient was then calculated (Table 2). The CXR “global” score was calculated as the sum of the GGO and CO scores of each patient, with a maximum score of 36. Finally, each patient was classified as “normal”, “GGO prevalent”, “CO prevalent”, or “mixed” based on the prevalent CXR pattern [14].

### 2.6. Ultrasound Evaluation

A subset of 25 patients underwent bed-side LUS. The examination was conducted with a portable MyLab^TM^ 25 Gold ultrasound unit (Esaote, Genova, Italy) and a dedicated CA631 convex transducer (range of frequency 1–8 mHz). We used low frequency and a single-focal modality at the pleural line. The depth was arranged on 6–7 cm and the harmonic-imaging system was deactivated. The LUS score was calculated across 12 chest zones (six on each hemithorax) using a scale from 0 (normal pattern, A-lines or non-significant B-lines), 1 (significant B-lines ≥3 per rib space), and 2 (coalescent B-lines with or without small consolidations) to 3 (consolidation), as previously reported [15]. A final “US global score” was calculated for each patient with a maximum score of 36.

### 2.7. Statistical Analysis

Categorical variables were described as absolute (*n*) and relative values (%), whereas continuous variables were described as median and range. To compare demographic data and baseline clinical characteristics between LIMC and HIMC groups, Chi square test and Fisher’s exact test for categorical variables and Mann–Whitney U test for continuous variables were used, as appropriate. The correlation between CXR global score and pO_2_, FiO_2_, P/F ratio on admission was assessed for the entire study population and in the LIMC and HIMC groups using the nonparametric Spearman’s rank method. Univariate logistic regression analysis, followed by a multivariate logistic regression, was performed to detect the strongest predictors of level of care. The covariates included in the final model were those that were significant in the univariate regression analyses. The correlation between LUS global score and the corresponding CXR global score and FiO_2_ was calculated using the nonparametric Spearman’s rank method.

All data were analyzed using SPSS Software version 25.0 (US: IBM Corp., New York, NY, USA). *p*-values < 0.05 were considered statistically significant. The graphs were obtained using the statistical package GraphPad Prism 7.0 (GraphPad Software, Inc., La Jolla, San Diego, CA, USA).

## 3. Results

### 3.1. Patient Demographics and Clinical Characteristics at Baseline

Demographic and clinical characteristics at baseline (i.e., on hospital admission) are summarized in Table 1.

Most patients were male (73%) with a median age on admission of 68 years. Seventy-one patients required LIMC during hospitalization and thirty-one HIMC. Patients requiring HIMC (HIMCs) were mainly male (87 vs. 67%; *p* = 0.05) and older [74 (28–85) vs. 63 (22–94) years; *p* = 0.03], with a higher body mass index (BMI) [31 (21–43) vs. 24 (16–31) kg/m^2^; *p* = 0.02]. Moreover, they had a heavier smoking history (10 (0–60) vs. 0 (0–60) pack/year (py); *p* = 0.01) and were mainly former smokers (61%). The most common presenting symptoms were fever (92%), cough (61%) and shortness of breath (34%), and with 5% of patients complaining of impaired sensory. The frequency of these symptoms did not differ between HIMCs and LIMCs. Interestingly, although the time interval between onset of respiratory symptoms and admission to the emergency unit was similar, HIMCs showed a greater impairment of respiratory gas exchange with a lower pO_2_ on room air on admission (60 (21–90) vs. 90 (54–119) mmHg; *p* < 0.0001), greater FiO_2_ requirement at the time of admission (39 (21–100) vs. 21 (21–51) %; *p* < 0.001) and worse P/F (158 (33–429) vs. 429 (106–567); *p* < 0.0001) compared to LIMCs. In the overall population, CVDs were the most frequent comorbidities (55%) that we observed. Among the metabolic comorbidities, diabetes mellitus was the most prevalent (26%), followed by obesity (8%) and dyslipidemia (13%). Hypothyroidism was the most frequent condition among the autoimmune diseases (3%). Oncologic diseases (13%) were equally distributed among organs affected (i.e., lung, prostate, pancreas. breast, colon). HIMCs reported more comorbidities, in particular cardiovascular diseases (CVDs) (80 vs. 49% of cases; *p* = 0.002), metabolic diseases (61 vs. 37%; *p* = 0.002) and oncologic diseases (22 vs. 8%; *p* = 0.05). Furthermore, this patient group showed a higher frequency of bacterial co-infections (42 vs. 15%; *p* = 0.002) during hospitalization. Finally, the hospitalization time was significantly longer for HIMCs compared to LIMCs [26 (7–119) vs. 8 (2–50) days; *p* < 0.0001], with 4 patients dying among HIMCs and only one among LIMCs (*p* = 0.01).

### 3.2. Radiological Features on Admission

On admission, HIMCs showed a more severe radiological impairment compared to LIMCs, with higher X-ray global score [8 (0–35) vs. 3 (0–22); *p* < 0.0001], GGO score (5 (0–15) vs. 1 (0–18); *p* < 0.001) and CO score (0 (0–35) vs. 0 (0–10); *p* = 0.02), respectively. When considering the prevalent CXR pattern, only one patient among HIMCs had a normal CXR on admission compared to LIMCs (14; *p* = 0.003), with similar proportion of patients with “GGO prevalent” and “CO prevalent” patterns in the HIMC and LIMC groups.

### 3.3. Radiological Correlations

**Figure 1:**
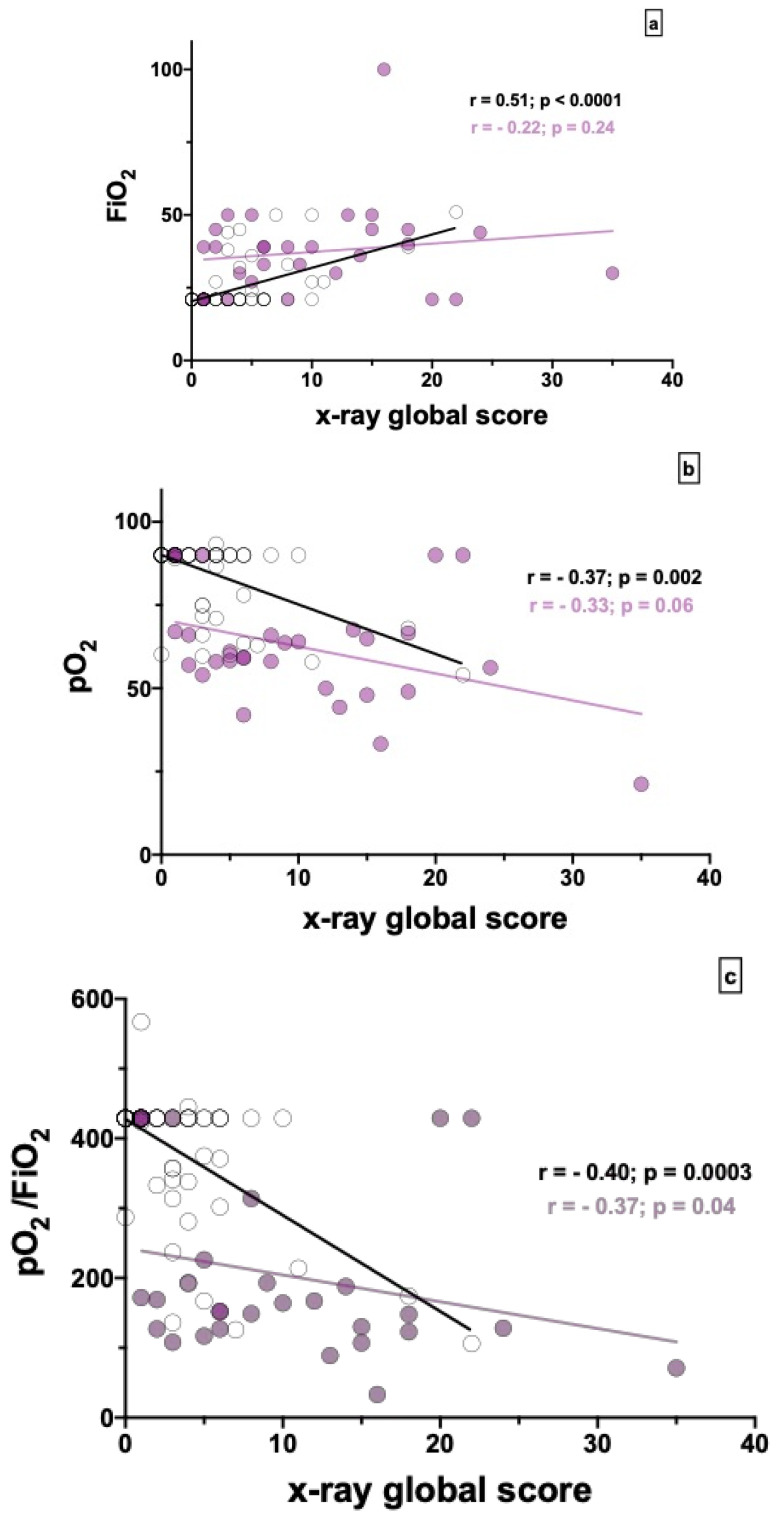
Correlation between chest x-ray global score and (**a**) FiO2 at admission, (**b**) pO_2_ at admission in room air, and (**c**) pO_2_/FiO_2_ at admission in room air in the study population categorized in LIMC and HIMC groups. Black points indicate LIMC patients and purple points indicate HIMC patients.

In the overall study population, a positive correlation was observed between CXR global score and FiO_2_ on admission (*r* = 0.6, *p* < 0.0001). When stratified by level of care, the correlation between CXR global score and FiO_2_ on admission was confirmed in LIMCs (*r* = 0.51, *p* < 0.0001) but not in HIMC (Figure 1a). In the overall study population, we observed a negative correlation between CXR global score and pO_2_ on admission (*r* = −0.6, *p* < 0.0001). When stratified by level of care, the correlation between CXR global score and pO_2_ on admission was confirmed in LIMCs (*r* = −0.37; *p* = 0.02) but not in HIMCs (Figure 1b). Finally, in the overall study population, we observed a negative correlation between CXR global score and P/F on admission (*r* = −0.6, *p* < 0.0001). When stratified by level of care, the correlation between CXR global score and P/F at admission was confirmed in both LIMCs (*r* = −0.40; *p* = 0.0003) and HIMCs (*r* =−0.37; *p* = 0.04) (Figure 1c).

### 3.4. Predictors of Level of Care Requirement

Univariate logistic regression analysis of factors associated with level of care revealed that sex, age, smoking history, FiO_2_, pO_2_ in room air at admission, bacterial co-infections developed during hospitalization, CVDs, metabolic and oncologic diseases and chest X-ray global score had significant positive association with a higher level of care in the entire study population (Table 3). Multivariate analysis performed using variables with statistical significance in univariate analysis revealed that smoking history (Odds ratio 6.55; 95% CI: 1.15–52.09; *p* = 0.04), pO_2_ (36.7, 3.64–681.4; *p* = 0.005), CVDs (10.89, 1.44–112; *p* = 0.02), and oncologic diseases (17.13, 1.76–242.6; *p* = 0.02) were independent predictors of higher level of care in patients with SARS-CoV-2 infection.

### 3.5. Ultrasound Evaluation

**Figure 2:**
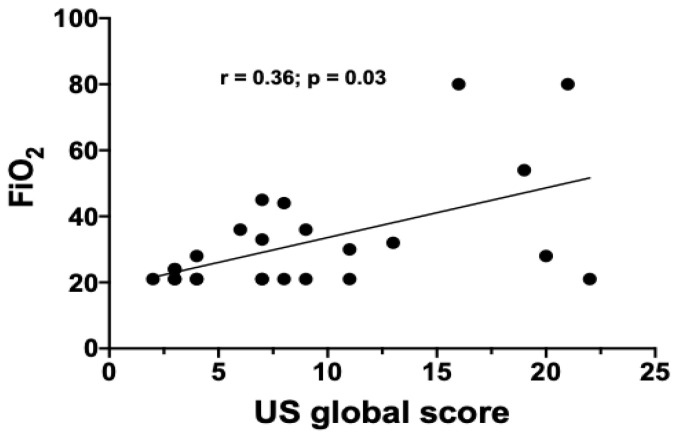
Correlation between lung ultrasound (US) global score and FiO_2_ in the subgroup of patients undergoing US examination.

**Figure 3:**
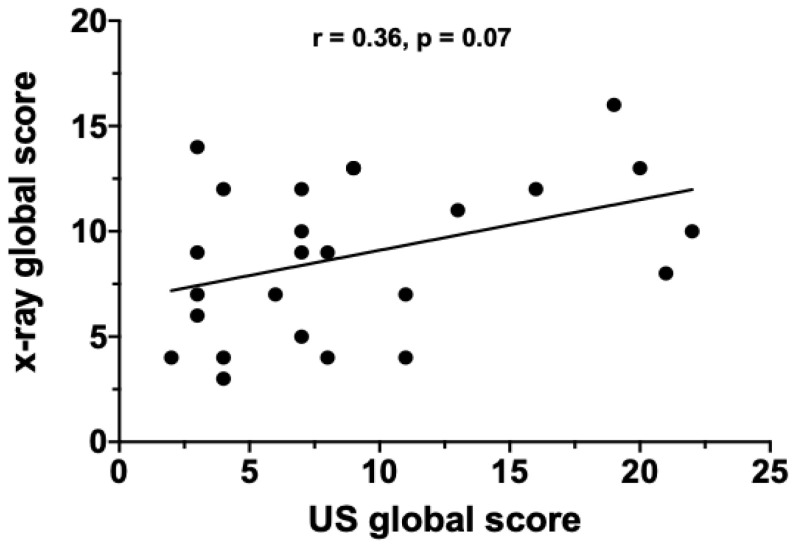
Correlation between lung US global score and X-ray global score in the overall study population.

**Figure 4:**
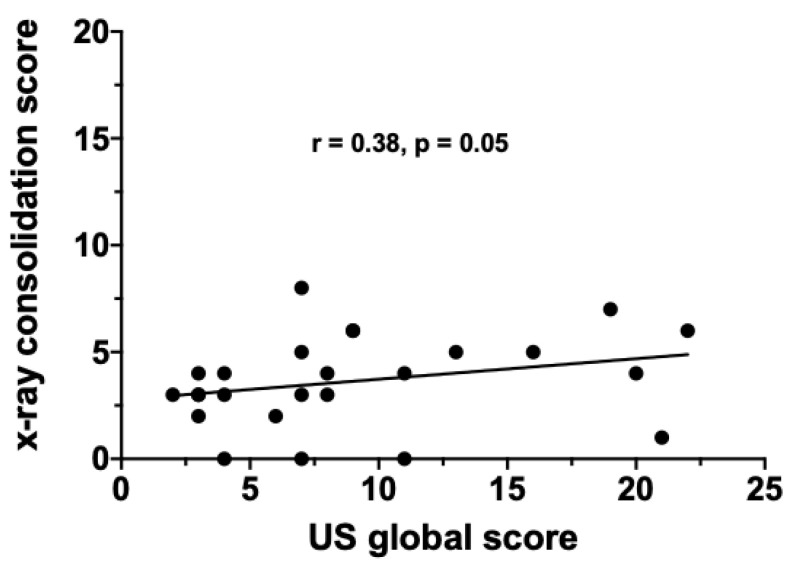
Correlation between chest X-ray consolidation score and US global score in the overall study population.

A subset of 25 patients underwent a bed-side LUS after a median time of 11 days from admission. In parallel, CXRs were performed in the same patients at the same time point. The median LUS global score was 7 (2–22), whereas the median CXR global score was 9 (3–13). The LUS global score positively correlated with the FiO_2_ requirement at the time of the US examination (*r* = 0.36; *p* = 0.03) (Figure 2). Conversely, the correlation between LUS global score and CXR global score only trended towards statistical significance (*r* = 0.36, *p* = 0.07) (Figure 3). Finally, the LUS global score positively correlated with the CXR CO score (*r* = 0.38; *p* = 0.05) (Figure 4) but not with the GGO score.

## 4. Discussion

This is a retrospective analysis of clinical features and radiographic severity scores in patients with COVID-19 and how these parameters on hospital admission correlate with different levels of medical care (i.e., HIMC vs. LIMC). A subgroup of patients also underwent LUS, which was correlated with chest radiographs. Our study revealed that patients with COVID-19 who required a HIMC are mainly men, former smokers with a higher pack/year of smoking history, older and with a higher BMI compared to patients requiring LIMC. Furthermore, the majority of them reported at least one comorbidity (i.e., cardiovascular, metabolic, or oncologic) and required on emergency room oxygen supplementation due to low alveolar oxygen partial pressure (PaO_2_). Moreover, using a multivariate analysis, we found that a heavier smoking history, pO_2_ level on room air, and presence of cardiovascular or oncological disease on admission were independent predictors of the need of HIMC.

Our findings mirror those from previous studies indicating that older male patients with comorbidities are at higher risk of pulmonary infection and fatal consequences from COVID-19 [16,17]. In our study, we show that the number of pack-years was significantly higher in former smokers who required intensive care compared to those requiring LIMC. Moreover, the proportion of former smokers was markedly increased among severe patients, whereas nonsmokers with COVID-19 experienced a milder illness, which required low-flow oxygen supplementation. This is in line with other reports that explored the association between smoking and progression of COVID-19 pneumonia [18]. Notably, in our study, multivariate analysis revealed that smoking history was an independent risk factor for HIMC. We speculate that cigarette smoke upregulates the expression of angiotensin-converting enzyme 2 receptors, which in turn facilitate SARS-CoV-2 entry in the respiratory epithelium; this implies that smoking habit may represent a risk factor for developing severe illness even among former smokers. In other words, having quit smoking does not seem to prevent the risk of severe COVID-19 pneumonia [19]. Chronic respiratory disease, including, among others, Chronic Obstructive Pulmonary Disease (COPD), carry a worse prognosis when associated with chronic conditions, such as cardiovascular diseases [20,21,22]. Interestingly, in our cohort, concomitant CVDs and neoplasms were independent risk factors for hospitalization in HIMC, with up to 80% of patients who required HIMC reporting an history of CVD (mainly arterial hypertension). A recent meta-analysis of 1576 patients concluded that hypertension, chronic respiratory disease and CVD are risk factors for severe COVID-19 disease [23]. Considering our study population, we observed that CVDs are the most frequent comorbidities (55% of cases), 27% of patients suffered from diabetes mellitus, 13% showed blood tests positive for dyslipidemia, and 8% of our patients were obese. We, therefore, are in line with an Italian nationwide observational study of COVID-19 inpatients which reported a linear direct relationship between the number of comorbidities and the risk of death [24].

All these findings emphasize the importance of past medical history and comorbidities in the disease course of COVID-19 patients, as they may predispose to worse outcome and higher intensity of care. pPO_2_ level < 90 mmHg on admission to emergency room was an additional independent predictor of HIMC requirement. This is interesting, as the duration of symptoms (i.e., median of 4 days) did not differ between patients requiring HIMC and patients requiring LIMC. Thirty-one subjects required subsequent admission to ICU due to worsening of pneumonia and gas exchange. On admission, these patients displayed extensive radiological impairment in terms of both GGO score and consolidation. In the overall population gas exchange parameters correlate significantly with radiological scores but, interestingly enough, this correlation was mainly due to patients who remained in the LIMC group. Indeed, in this group, radiological score correlated negatively with pPO_2_ levels and positively with FiO_2_ reflecting exact correspondence between respiratory failure and radiologic impairment. Conversely, among patients who subsequently required HIMC, CXR at baseline showed a variety of radiologic impairment, ranging from normal to highly abnormal however without a concurrent relation with gas abnormality. This result might arise attention to that patients who display discrepancies between gas exchange parameters and CXR.

Pevious reports on CXR findings in COVID-19 patients focused on the distribution and type of lung abnormalities. Wong and coauthors demonstrated that CXR at baseline has a sensitivity of 69% for a diagnosis of COVID-19 pneumonia, corroborating the utility of CXR in the initial evaluation of subjects with suspected COVID-19 pneumonia, thus obviating the need for CT [6]. Toussie and colleagues have recently reported that initial CXR severity score is also an independent predictor of outcome in COVID-19 patients [3]. We could not replicate this finding, but our study population was older than that studied by Toussie et al. The prognostic role of CXR in COVID-19 pneumonia therefore needs to be clarified in larger studies.

Lung ultrasound has been suggested as a potential diagnostic tool for COVID-19 pneumonia given the predominant involvement of the lung periphery [7]; lung ultrasound is a relatively simple technique that can be easily applied at patient bedside [25]. In our study, we investigated its role in the late phase of COVID-19 pneumonia and its relation with CXR in a subgroup of patients hospitalized in a low-intensity care setting. We found a significant correlation between LUS features and FiO_2_ level, suggesting these two parameters can be integrated into the evaluation of patients with COVID-19 pneumonia. LUS global score positively correlated with CXR consolidation score while the correlation with CXR global score only trended towards statistical significance. Although only exploratory, these findings may anticipate further studies mainly focused on the utility of LUS as a monitoring tool, possibly limiting the use of serial CXR, at least in the advanced phase of COVID-19 pneumonia. In this regard, LUS has been suggested as a potential substitute for CXR in the follow-up of various lung diseases in ICU [26], reducing the number of CXRs performed and relative medical costs without affecting patient outcome. Of interest in a recent study by Møller-Sørensen and colleagues, the usefulness of bed-side LUS in ICU patients treated with extracorporeal membrane oxygenation (ECMO) was assessed during the COVID-19 pandemic. Authors used a three-zone score for each lung (anterior, posterior and lateral) with a maximum of 102 points for patient. LUS score demonstrated a strong correlation with compliance during mechanical ventilation. Moreover, a lower LUS score advanced weaning capacity from ECMO [27]. Soldati and colleagues have also suggested that LUS can be useful in COVID-19 pneumonia by identifying disease extension and specific patterns, as well as their evolution toward the consolidation phase [28], thus providing further support to the role of LUS in the follow-up of patients with COVID-19 pneumonia. At present, however, the majority of studies performed during the COVID-19 pandemic focused on ultrasonographic signs and disease patterns at presentation rather than overtime [29,30,31,32,33]. Accordingly, the role of LUS in monitoring the evolution of COVID-19 pneumonia needs to be confirmed in larger studies.

The results of our study should be interpreted in the light of important limitations. First, this is not a longitudinal study and we retrospectively collected all clinical and radiological data; therefore, the accuracy of the clinical information depends on medical records, which may introduce inaccuracies. However, every effort was made to limit this risk, even asking to the patients to fill all the missing data when possible. Second, the study population was relatively small, particularly the subset of patients for whom LUS data were available, although this was an exploratory analysis, and its findings should be viewed as such. Clearly, these data need to be validated in larger, independent, prospectively collected populations of patients.

In summary, our study identified clinical features that strongly predict the level of medical setting required by patients with COVID-19 pneumonia (HIMC or LIMC). These findings allow the identification of patients at risk for severe disease and worse outcome already on hospital admission. The correlation of LUS with clinical parameters and radiological score provides the basis for future studies on the utility of LUS in the follow-up of patients with COVID-19 pneumonia.

## Figures and Tables

**Table 1:** Baseline demographics and clinical features of the overall population hospitalized for severe acute respiratory syndrome coronavirus type 2 (SARS-CoV-2) related infection, as well as of the two subgroups categorized in low (LIMC) and high (HIMC) intensity medical care.

	Overall Population	Low-Intensity Medical Care (LIMC)	High-Intensity Medical Care (HIMC)	*p* Value
	(*n* = 102)	(*n* = 71)	(*n* = 31)	
Male—*n* (%)	75 (73)	48 (67)	27 (87)	0.05
Age at admission—years	68 (22–94)	63 (22–94)	74 (28–85)	0.03
Smoking history—pack years	0 (0–60)	0 (0–60)	10 (0–60)	0.01
Current—*n* (%)	9 (9)	8 (11)	1 (3)	0.18
Former—*n* (%)	43 (42)	24 (34)	19 (61)	0.009
Nonsmokers—*n* (%)	50 (49)	41 (57)	9 (29)	0.007
BMI (kg/m^2^)	25 (16–43)	24 (16–31)	31 (21–43)	0.02
Lag time symptoms—diagnosis—days	4 (−4–23)	3 (−4–23)	6 (−2–22)	0.07
FiO_2_ at admission (room air)—%	21 (21–100)	21 (21–51)	39 (21–100)	<0.0001
pO_2_ at admission (room air)—mmHg	90 (21.2–119)	90 (54–119)	60 (21–90)	<0.0001
P/F at admission—value	429 (33–567)	429 (106–567)	158 (33–429)	<0.0001
Hospitalization—days	10.5 (2–119)	8 (2–50)	26 (7–119)	<0.0001
Bacterial co-infections—*n* (%)	24 (23)	11 (15)	13 (42)	0.002
Comorbidities				
CVD—*n* (%)	60 (59)	35 (49)	25 (80)	0.002
Respiratory diseases—*n* (%)	18 (18)	11 (15)	7 (22)	0.39
Autoimmune diseases—*n* (%)	12 (12)	10 (14)	2 (6)	0.34
Metabolic diseases—*n* (%)	45 (44)	26 (37)	19 (61)	0.002
Oncologic—*n* (%)	13 (13)	6 (8)	7 (22)	0.05
Death—*n* (%)	6 (6)	1 (1)	4 (13)	0.01

Values are expressed as numbers and (%) or median and range, as appropriate. Negative values refer to patients with symptoms occurring after admission to the hospital. To compare demographic between LIMC and HIMC, Chi square test and Fisher *t* test (*n* < 5) for categorical variables and Mann–Whitney *t* test for continuous variables were used.

**Table 2:** Baseline radiological scores of the overall population hospitalized for SARS-CoV-2 related infection, and of the two subgroups categorized in low (LIMC) and high (HIMC) intensity medical care.

	Overall Population	Low-Intensity Medical Care (LIMC)	High-Intensity Medical Care (HIMC)	*p* Value
	(*n* = 102)	(*n* = 71)	(*n* = 31)	
X-ray global score (GGO + consolidations)	3 (0–35)	3 (0–22)	8 (0–35)	<0.0001
GGO—score	2 (0–18)	1 (0–18)	5 (0–15)	<0.0001
Consolidation—score	0 (0–35)	0 (0–10)	0 (0–35)	0.02
Normal—*n* (%)	15 (15)	14 (20)	1 (3)	0.003
GGO prevalent—*n* (%)	66 (65)	44 (62)	22 (71)	0.38
Consolidation prevalent—*n* (%)	15 (15)	11 (16)	4 (13)	0.73
Mixed—*n* (%)	6 (6)	2 (3)	4 (13)	0.04

Values are expressed as numbers and (%) or median and range as appropriate. To compare demographic data and baseline clinical characteristic between LIMC and HIMC, Chi square test and Fisher *t* test (*n* < 5) for categorical variables and Mann–Whitney *t* test for continuous variables were used.

**Table 3:** Predictive factors of higher level of care in the overall population of patients hospitalized for COVID related infection.

	Univariate Analysis		Multivariate Analysis	
	OR (95% IC)	*p*	OR (95% IC)	*p*
Sex (male vs. female)	3.23 (1.01–11.89)	0.04	0.54 (0.06–4.22)	0.55
Age (yr, ≥ 68 vs. < 68)	3.34 (1.38–8.61)	0.009	0.51 (0.06–3.03)	0.49
Smoking history (p/y, > 0 vs. ≤ 0)	2.72 (1.08–7.27)	0.03	6.55 (1.15–52.09)	0.04
FiO_2_ at admission (%, > 21 vs. ≤ 21)	13.1 (4.92–39.2)	<0.0001	4.17 (0.60–29.89)	0.14
pO_2_ at admission (room air) (mmHg, < 90, ≥ 90)	13 (4.78–40.4)	<0.0001	36.7 (3.64–681.4)	0.005
Lag time symptoms—diagnosis—(days, ≥ 4 vs. < 4)	2.18 (0.90–5.50)	0.08	–	–
P/F at admission (≥ 429 vs. < 429)	9.60 (3.59–29.26)	<0.0001	16.61 (3.34–128.3)	0.002
Bacterial co-infections (yes vs. no)	4.64 (1.75–12.72)	0.002	2.48 (0.38–17.78)	0.34
CVDs—(yes vs. no)	5.14 (1.89–16.6)	0.002	10.89 (1.44–112.0)	0.02
Respiratory diseases—(yes vs. no)	5.14 (1.89–16.6)	0.34	–	–
Autoimmune diseases—(yes vs. no)	0.43 (0.06–1.79)	0.30	–	–
Metabolic diseases—(yes vs. no)	2.99 (1.25–7.44)	0.01	2.63 (0.54–14.76)	0.24
Oncologic diseases—(yes vs. no)	3.29 (1.00–11.25)	0.04	17.13 (1.76–242.6)	0.02
X-ray global score (> 3 vs. < 3)	3.33 (1.32–9.29)	0.01	0.40 (0.02–3.63)	0.43

Values are expressed as odds ratio (95% confidence interval). Logistic regression analysis in relation to level of care was used to determine the relationship of clinical and radiological characteristics with higher level of care needed during hospitalization.

## Abbreviations

COVID-19	Coronavirus disease 2019
SARS-CoV-2	Severe acute respiratory syndrome coronavirus type 2
ICU	Intensive care unit
CT	computed tomography
CXR	chest X-ray
US	ultrasound
HFNC	high-flow nasal cannula
LIMC	low-intensity medical care
HIMC	high-intensity medical care
GGO—	ground glass opacities
CO	consolidations
CVD	cardiovascular diseases

## References

[1] Lavezzo E., Franchin E., Ciavarella C., Cuomo-Dannenburg G., Barzon L., Del Vecchio C., Rossi L., Manganelli R., Loregian A., Imperial College COVID-19 Response Team (2020). Suppression of a SARS-CoV-2 outbreak in the Italian municipality of Vo’. Nature.

[2] Litmanovich D.E., Chung M., Kirkbride R.R., Kicska G., Kanne J.P. (2020). Review of Chest Radiograph Findings of COVID-19 Pneumonia and Suggested Reporting Language. J. Thorac. Imaging.

[3] Toussie D., Voutsinas N., Finkelstein M., Cedillo M.A., Manna S., Maron S.Z., Jacobi A., Chung M., Bernheim A., Eber C. (2020). Clinical and Chest Radiography Features Determine Patient Outcomes In Young and Middle Age Adults with COVID-19. Radiology.

[4] Minns F.C., Mhuineachain A.N., Van Beek E.J.R., Ritchie G., Hill A., Murchison J.T. (2015). Presenting CXR Phenotype of H1N1. Flu Compared with Contemporaneous Non-H1N1, Community Acquired Pneumonia, during Pandemic and Post-Pandemic Outbreaks’. Eur. J. Radiol..

[5] Cozzi D., Albanesi M., Cavigli E., Moroni C., Bindi A., Luvarà S., Lucarini S., Busoni S., Mazzoni L.N., Miele V. (2020). Chest X-ray in new Coronavirus Disease 2019 (COVID-19) infection: Findings and correlation with clinical outcome. Radiol. Med..

[6] Wong H.Y.F., Lam H.Y.S., Fong A.H.T., Leung S.T., Chin T.W.Y., Lo C.S.Y., Lee E.Y.P. (2020). Frequency and Distribution of Chest Radiographic Findings in COVID-19 Positive Patients Authors. Radiology.

[7] Rubin G.D., Ryerson C.J., Haramati L.B., Sverzellati N., Kanne J.P., Raoof S., Schluger N.W., Volpi A., Yim J.-J., Martin I.B. (2020). The Role of Chest Imaging in Patient Management During the COVID-19 Pandemic. Chest.

[8] Orso D., Guglielmo N., Copetti R. (2018). Lung ultrasound in diagnosing pneumonia in the emergency department: A Systematic Review and Meta-Analysis. Eur. J. Emerg. Med..

[9] Gargani L., Soliman-Aboumarie H., Volpicelli G., Corradi F., Pastore M.C., Cameli M. (2020). Why, when, and how to use lung ultrasound during the COVID-19 pandemic: Enthusiasm and caution. Eur. Hear. J. Cardiovasc. Imaging.

[10] Pierce C.W. (2020). Clarifying the role of lung ultrasonography in COVID-19 respiratory disease. Can. Med. Assoc. J..

[11] See K.C., Ong V., Tan Y.L., Sahagun J., Taculod J. (2018). Chest radiography versus lung ultrasound for identification of acute respiratory distress syndrome: A retrospective observational study. Crit. Care.

[12] Borghesi A., Maroldi R. (2020). COVID-19 outbreak in Italy: Experimental chest X-ray scoring system for quantifying and monitoring disease progression. Radiol. Med..

[13] Nicolini A., Ferrera L., Rao F., Senarega R., Ferrari-Bravo M. (2012). Chest radiological findings of influenza A H1N1 pneumonia. Rev. Port. Pneumol..

[14] Giraudo C., Cavaliere A., Fichera G. (2020). Validation of a composed COVID-19 chest x-rAy scoRE: The CARE project. Eur. Respir. J. Open Res..

[15] Smith M.J., Hayward S.A., Innes S., Miller A.S.C. (2020). Point-of-Care Lung Ultrasound in Patients with COVID-19 –a Narrative Review. Anaesthesia.

[16] Chen N., Zhou M., Dong X., Qu J., Gong F., Han Y., Qiu Y., Wang J., Liu Y., Wei Y. (2020). Epidemiological and Clinical Characteristics of 99 Cases of 2019-Novel Coronavirus (2019-nCoV) Pneumonia in Wuhan, China. SSRN Electron. J..

[17] Yang X., Yu Y., Xu J., Shu H., Xia J., Liu H., Wu Y., Zhang L., Yu Z., Fang M. (2020). Clinical course and outcomes of critically ill patients with SARS-CoV-2 pneumonia in Wuhan, China: A single-centered, retrospective, observational study. Lancet Respir. Med..

[18] Vardavas C.I., Nikitara K. (2020). COVID-19 and smoking: A systematic review of the evidence. Tob. Induc. Dis..

[19] Smith J.C., Sausville E.L., Girish V., Lou Yuan M., Vasudevan A., John M.K., Sheltzer J.M. (2020). Cigarette Smoke Exposure and Inflammatory Signaling Increase the Expression of the SARS-CoV-2 Receptor ACE2 in the Respiratory Tract. Dev. Cell.

[20] Huiart L., Ernst P., Suissa S. (2005). Cardiovascular Morbidity and Mortality in COPD. Chest.

[21] Curkendall S.M., Lanes S., De Luise C., Stang M.R., Jones J., She D., Goehring E. (2006). Chronic obstructive pulmonary disease severity and cardiovascular outcomes. Eur. J. Epidemiol..

[22] Gan W.Q., Man S.F.P., Senthilselvan A., Sin D.D. (2004). Association between chronic obstructive pulmonary disease and systemic inflammation: A systematic review and a meta-analysis. Thorax.

[23] Yang J., Zheng Y., Gou X., Pu K., Chen Z., Guo Q., Ji R., Wang H., Wang Y., Zhou Y. (2020). Prevalence of Comorbidities and Its Effects in Coronavirus Disease 2019 Patients: A Systematic Review and Meta-Analysis. Int. J. Infect. Dis..

[24] Polverino F., Stern D.A., Ruocco G. (2020). Comorbidities, cardiovascular therapies and COVID-19 Mortality: A Nationwide, Italian Observational Study (ItaliCO). Front. Cardiovasc. Med..

[25] Soldati G., Demi M. (2017). The use of lung ultrasound images for the differential diagnosis of pulmonary and cardiac interstitial pathology. J. Ultrasound.

[26] Brogi E., Bignami E., Sidoti A., Shawar M., Gargani L., Vetrugno L., Volpicielli G., Forfori F. (2017). Could the Use of Bedside Lung Ultrasound Reduce the Number of Chest X-Rays in the Intensive Care Unit?. Cardiovasc. Ultrasound.

[27] Møller-Sørensen H., Gjedsted J., Jørgensen V.L., Hansen K.L. (2020). COVID-19 Assessment with Bedside Lung Ultrasound in a Population of Intensive Care Patients Treated with Mechanical Ventilation and ECMO. Diagnostics.

[28] Soldati G., Smargiassi A., Inchingolo R., Buonsenso D., Perrone T., Briganti D.F., Perlini S., Torri E., Mariani A., Mossolani E.E. (2020). Is There a Role for Lung Ultrasound During the COVID -19 Pandemic?. J. Ultrasound Med..

[29] Volpicelli G., Gargani L. (2020). Sonographic signs and patterns of COVID-19 pneumonia. Ultrasound J..

[30] Huang Y., Wang S., Liu Y., Zhang Y., Zheng C., Zheng Y., Zhang C., Min W., Zhou H., Yu M. (2020). A Preliminary Study on the Ultrasonic Manifestations of Peripulmonary Lesions of Non-Critical Novel Coronavirus Pneumonia (COVID-19). SSRN Electron. J..

[31] Nazerian P., Cerini G., Vanni S., Gigli C., Zanobetti M., Bartolucci M., Grifoni S., Volpicelli G. (2016). Diagnostic accuracy of lung ultrasonography combined with procalcitonin for the diagnosis of pneumonia: A pilot study. Crit. Ultrasound J..

[32] Poggiali E., Dacrema A., Bastoni D., Tinelli V., Demichele E., Ramos P., Marciano T., Silva M., Vercelli A., Magnacavallo A. (2020). Can Lung US Help Critical Care Clinicians in the Early Diagnosis of Novel Coronavirus (COVID-19) Pneumonia?. Radiology.

[33] Peng Q.Y., Wang X.T., Zhang L.N. (2020). Findings of Lung Ultrasonography of Novel Corona Virus Pneumonia during the 2019–2020 Epidemic. Intensive Care Med..

